# Personalizing health call center services: results from an online survey on user experiences in Kuwait

**DOI:** 10.12688/f1000research.170997.1

**Published:** 2026-03-05

**Authors:** Elham Aldousari, Maha Alhajeri, Dennis Kithinji

**Affiliations:** 1 Health Informatics and Information Management, Kuwait University College of Allied Health Sciences, Kuwait City, Al Asimah Governate, 90805, Kuwait; 2 Research and Writing, Medright Consulting LTD, Maua, Meru, 60600, Kenya; 3 Medical Laboratory Sciences, Meru University of Science and Technology, Meru, Meru County, 60200, Kenya

**Keywords:** call center; telehealth; remote care; personal satisfaction; user experience; communication; educational status; Kuwait

## Abstract

**Background:**

Limited evidence exists regarding user satisfaction with 151 call center service established by the Ministry of Health in Kuwait. This study explores user satisfaction with a healthcare call center by analyzing user experiences with Kuwait’s Ministry of Health 151 call center.

**Method:**

A national anonymous online survey was conducted through Twitter, WhatsApp, and Facebook. A 5-point Likert scale was used to obtain user ratings of their satisfaction with the various aspects of the 151 call center. Mean ratings and their standard deviations summarized the ratings, as One-way ANOVA tested the existence of statistically significant differences in mean ratings across groups to identify factors influencing the ratings.

**Results:**

Out of 1020 respondents, 28.4% (n = 290) had contacted the 151 service with vaccination-related inquiries, general inquiries, and COVID-19-related inquiries. Overall, female participants expressed statistically significantly higher satisfaction levels with quality (p < 0.021) and communication with doctors (p < 0.030). Response to calls, forwarding of complains, and representatives’ knowledge and skills were rated highly by respondents with high school diploma and bachelor’s degree compared to individuals with less than high school education, master’s degree, and doctorate degree (p = 0.032, p = 0.012, and p = 0.007, respectively).

**Conclusions:**

Nationally-representative Kuwaiti embraced the 151 call center, with gender and education level influencing the participants’ satisfaction with 151. The findings revealed the need for the call center to tailor-make services by gender and education diversities to optimize user experiences.

## Introduction

The evolution of the healthcare system towards digitization has modelled the efficiency of the contemporary care matrix around call centres as pivotal communication points. According to Gordon et al., ^1^ call centres are patients’ first point of contact, shaping their initial impressions, influencing satisfaction, and improving patient access, retention, and service quality. ^2^ For example, directing patients to appropriate services early reduces hospital readmissions significantly. ^3^ Also, the appointment calendars created at call centres enhance the management of appointments, increase internal referrals, and optimize physician availability, which minimize wasted clinical time. ^4^ On the other hand, inefficiencies in call centres can lead to misdirected care, higher patient readmissions, and skyrocketing healthcare costs.

Many healthcare systems worldwide are grappling with the rapid landscape evolution towards digitization. Nunes and Ferreira ^5^ posit that digitization has transformed traditional call centres into comprehensive contact points that handle phone calls and manage emails, text messages, and digital health records. However, as patients increasingly rely on these centres for immediate responses and convenient remote access to medical information, many medical call centres lack the resources to handle the complexity and volume of modern patient inquiries. ^6^ Hence, the context presents a care gap, showing the unpreparedness of health systems to adapt to evolving patient and healthcare landscapes. Mehta, Pandit, and Kulkarni ^7^ noted that modern healthcare systems should meet the rising demands linked to digitization and the evolving patient population, with targeted initiatives for recruiting skilled personnel capable of navigating the clinical and technical domains of the contemporary healthcare landscape. ^4, 8^ Hence, digitization initiatives should balance priorities between quality and efficiency of services.

The 151-call center, run by Kuwait’s Ministry of Health, ^9^ is an inbound service where patients call to ask questions, make appointments, or speak with medical experts regarding contemporary care issues. Most healthcare call centers in Kuwait deal with patients or their families, who frequently contact these centers during emergencies or under stress. ^10^ Hence, users anticipate prompt, succinct, and reliable responses when contacting Kuwait’s healthcare call centers. However, the overarching challenges reported in contemporary healthcare call centers revolve around long wait times and unclear information, leading to caller frustration and high abandonment rates, ^11, 12^ which has been a notable challenge in Kuwait. Yet, limited published evidence is available on the operational efficiency of Kuwait’s 151 call service regarding user satisfaction with service quality and communication effectiveness. This study explores patient experiences with Kuwait’s Ministry of Health 151 call center regarding quality of call center services (CCS), employee skills (ES), communication with doctors (CWD), remote consultations, and utility of the 151 service, as well as the factors that could be influencing them.

## Methods

### Study design

This study used a cross-sectional study design and an online population survey approach.

### Study setting

The study was conducted across all governorates in Kuwait in August 2021. Users of the 151 call centre were anonymously recruited to rate it through a survey link shared on social media platforms in Kuwait.

### Participants

Only adults aged 18 years and above living in Kuwait were allowed to participate. The online survey included eligibility questions at the start that automatically exited the people who clicked the link but did not meet the eligibility criteria from the survey.

### Variables

The dependent variables were ratings of satisfaction with the quality of the 151 call center services, and communication with representatives and doctors. The independent variables were age, gender, governorate, educational level, marriage status, and nationality.

### Measurement: Study questionnaire and pretesting

The questionnaire used was designed applying the five-point Likert scale and administered using the Questionpro software. The 16-item questionnaire covered five domains: demographics, interaction with the call center, satisfaction with teleconsultation services, perceived advantages and information sources, and reasons for non-use. The questionnaire was translated back and forth from English to Arabic and then reviewed for accuracy by the original authors and a translator.

Content and construct validity of the questionnaire was established by getting feedback about it from two social science experts and two physicians from the Ministry of Health. Face validity and reliability of the questionnaire were tested during a pilot study that collected data from 35 participants who were excluded from the main study. Reliability assessment focused on satisfaction, skills, communication, and advantages factors. Cronbach’s alpha was calculated to measure the reliability of the questionnaire. Cronbach’s alpha corresponding for the satisfaction, skills, communication, and advantages subscales were 0.890, 0.937, 0.954, and 0.900, respectively.

### Data collection

The first page of the survey presented the prospective participants with an informed consent form and an option to click a “I agree to participate” button to progress to the survey questions if they consented or to click an “I choose to exit” button if they did not agree to participate. The online nature of the survey that was conducted during the COVID-19 pandemic and the intent to maintain total anonymity made signing physical consent forms or obtaining oral consent untenable. A statement that the survey targeted adults aged 18 years and above living in Kuwait was boldly placed on the first page of the survey to ensure only adults participated.

The survey link was shared online through social media platforms including Twitter, WhatsApp, and Facebook to reach adult Kuwaiti across the country. Skip logic was applied in Questionpro so that participants who self-reported having called 151 accessed questions in domains 1,2,3, and 4; while participants who self-reported having not called 151 accessed domains 1,2 (except the question regarding the purpose of the call), 4, and 5. The participants had the option to skip any question that they were not comfortable answering.

### Bias

Anonymizing the online survey reduced social desirability bias and interviewer bias since the issues of responses being linked to identities and responding to please the interviewer were inexistent. Recruiting participants from multiple social media platforms reduced sampling bias. Non-response bias was addressed by making the survey brief, mobile friendly, and allowing participants to only answer the questions they preferred. Validating and pretesting the questionnaire reduced measurement bias.

### Sample size estimation

The Cochran’s formula for sample size calculation was used to estimate the sample size since this study was a population survey. It estimated that 384 call center users were required to measure satisfaction with the call center at 95% confidence level. However, since addition of extra responses within the study period was feasible at negligible costs, as many respondents as could answer the survey within the study period were allowed to participate.

### Data analysis

The collected data were extracted from Questionpro into an Excel codebook for analysis. The data were analysed using IBM’s Statistical Package for Social Sciences 25.0. All the collected data were analysed since any feedback on a service could be constructive. Descriptive analyses included means and standard deviations for continuous variables and percentages for categorical variables. One-way ANOVA was used to examine whether satisfaction with call center services, employee skills, and doctor communication differed significantly across various groups of participants. Pairwise deletion was applied to address missing data, which ensured every feedback on the satisfaction with the individual aspects of the call center counted.

## Results

### Demographic characteristics

A total of 1,020 individuals, mainly females, completed the survey. Nearly 70% of them were under 30 years old. Three-quarters had at least a high school diploma. About 45% of the participants were living in Jahra and Farwaniya governorates in Kuwait ( Table 1).

**Table 1.** Demographic characteristics of the users of the 151 call center in Kuwait that rated their satisfaction with it.

Variables	n (%)
**Gender**	
Male	419 (41.1)
Female	601 (58.9)
**Age**	
Less than 18 years	29 (2.8)
18–24 years	529 (51.9)
25–29 years	172 (16.9)
30–39 years	161 (15.8)
40–49 years	75 (7.4)
50–59 years	38 (3.7)
Over 60 years	16 (1.6)
**Education**	
Less than high school	28 (2.7)
High school	322 (31.6)
Diploma	182 (17.8)
Bachelor’s	439 (43.0)
Master’s	23 (2.3)
Doctoral	25 (2.5)
Other	1 (0.1)
**Nationality**	
Kuwaiti	812 (79.6%)
Other nationality	116 (11.4%)
Undefined nationality	92 (9.0%)
**Marital status**	
Single	654 (64.1%)
Married	307 (30.1%)
Divorced	56 (5.5%)
Widowed	3 (0.3%)
**Governorate**	
Jahra	252 (24.7%)
Farwaniya	198 (19.4%)
Asemah	179 (17.5%)
Ahmadi	157 (15.4%)
Hawaii	134 (13.1%)
Mubarak Al-Kabeer	100 (9.8%)
**Total**	**1,020**

### Participant experiences and satisfaction with the 151 call centre system

**Figure 1.**
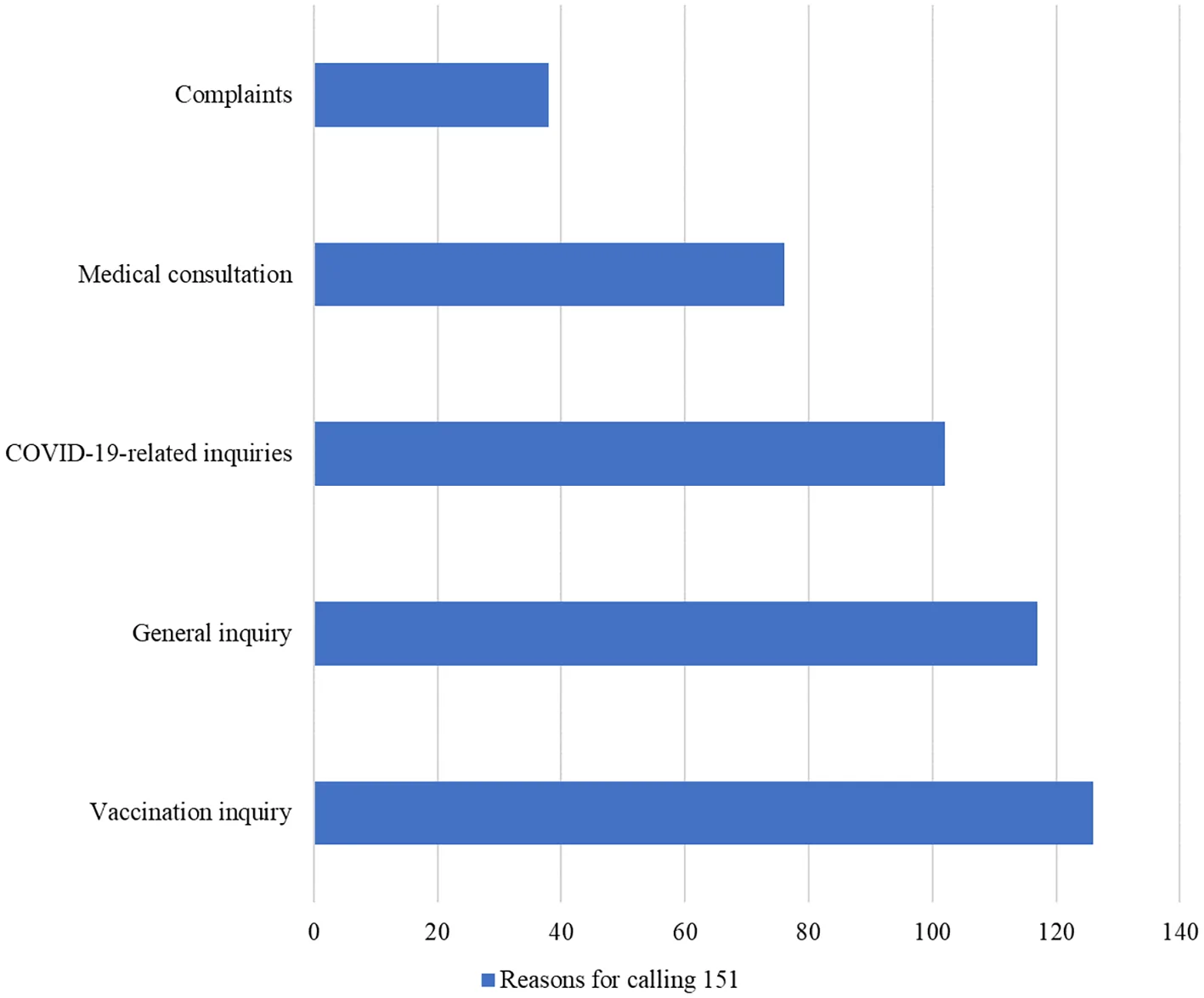
Numbers of participants that called 151 call center for various reasons.

All the 1020 participants responded to all the questions, 564 (55%) were aware of 151, and 730 had never called it. Callers had diverse reasons, mainly vaccination inquiries and general inquiries ( Figure 1). Reasons for not using 151 include low response to the call (n = 683), having no intention of using it (n = 608) or lacking awareness of the services it provided (n = 590). Furthermore, 504 participants stated they did not need it while 300 participants were unaware about it.

Participants were satisfied with the clarity of sound quality, explanation of the instructions, and the variety of the languages provided in the 151 call center system considering that the mean ratings for the respective subscales were 3.8, 3.5, and 3.5, respectively. However, they were dissatisfied with the speed of connection to the line (mean = 3.1, SD = 1.3). Participants tended to believe that the customer service representative was courteous, attentive, and understanding (mean ratings = 3.4–3.8, SD = 1.0–1.5).

Participants felt that doctors treated them respectfully, communicated with them clearly, listened to their complaints attentively, explained conditions clearly, and provided appropriate guidance (mean ratings = 3.5–3.7, SD = 1.1–1.2). However, their rating for having their issue resolved during the call was low (mean rating = 3.2, SD = 1.4) Nevertheless, the participants identified the 151 phone service as having several advantages including increasing health awareness, answering emergency inquiries, enhancing access to official and reliable sources of information, and meeting the medical information needs of the public since the participants rated the related statements 3.8 (SD = 1.1).

In all aspects except opening a quick line of communication and 24-hour operation, statistically significantly more females than males expressed satisfaction with the call center (p < 0.02). The differences in ratings were not practically significant since all the mean ratings were between 3 and 4 ( Table 2), which translates to neutrality to the statement. Similarly, female respondents statistically significantly rated the doctors better than male respondents (p < 0.03) but the differences were not practically significant (mean ratings = 3.4–3.7).

**Table 2.** Statistical differences between mean ratings of satisfaction with aspects of the 151 call center across demographic groups of the users.

Item	Categories	Demographic characteristic	Mean rating	Standard deviation	P value
Satisfaction with quality	Variety of language options	Male	3.4	1.1	0.013
		Female	3.7	1.0	
	Comprehensiveness of options	Male	3.1	1.2	0.021
		Female	3.4	1.1	
	Explanation of the instructions	Male	3.2	1.2	0.002
		Female	2.6	1.1	
	Clarity of sound quality	Male	3.6	1.1	0.009
		Female	3.9	0.9	
Communication with representatives	Quick response to the call	< high school education	1.8	1.3	0.032
		Some high school education	3.2	1.5	
		High school diploma	3.5	1.4	
		Bachelor’s degree	3.0	1.4	
		Master’s degree	2.5	1.4	
		Doctorate degree	2.4	1.1	
	Forwarding complaints	< high school education	2.4	0.9	0.012
		Some high school education	3.5	1.2	
		High school diploma	3.5	1.4	
		Bachelor’s degree	3.1	1.3	
		Master’s degree	2.8	1.2	
		Doctorate degree	2.3	1.1	
	Knowledgeable and skilled	< high school education	2.4	1.3	0.007
		Some high school education	3.5	1.2	
		High school diploma	3.7	1.3	
		Bachelor’s degree	3.3	1.3	
		Master’s degree	2.8	1.5	
		Doctorate degree	2.3	1.1	
Communication with doctors	Respectful	Male	3.5	1.1	0.016
		Female	3.8	1.1	
	Listening	Male	3.4	1.2	0.020
		Female	3.7	1.1	
	Language clarity and understandability	Male	3.5	1.1	0.030
		Female	3.8	1.1	
	Sufficiency of call for a consultation	Male	3.3	1.3	0.015
		Female	3.7	1.2	

Satisfaction with the courteousness of the customer service representative was mainly among participants whose level of education was a high school diploma (mean rating = 4.1, SD = 1.2) and some high school education (mean rating = 4.0, SD = 1.0). Participants with only primary education and those with advanced degrees were generally dissatisfied with knowledge and skills of the customer service representatives, promptness of response to the 151 calls, and forwarding of their inquiries to the relevant departments (mean ratings <2.8, SD < 1.5) ( Table 2).

## Discussion

Most of the participants who were aware of the 151 call center had enough knowledge and experience with it, and reported above-average satisfaction rates. The service received relatively high ratings for sound clarity, instruction clarity, and language diversity. User satisfaction in telemedicine is dependent on the technical soundness of the foundational concepts and the ease of platform accessibility, ^13^ hence the 151 call center service is technically sound and accessible. However, the speed of connection to the call centre’s services received the lowest satisfaction rating, indicating the possibility of an operational problem. Zinger et al. ^14^ also reported response delays as among the most frequent complaints among users of medical helplines similar to the 151 call center. Hence, in emergency cases, such helplines may not be optimally effective.

Most participants were satisfied with the skills of the employees operating Kuwait’s 151 call centres, identifying them with professionalism, communication, courteousness, attentiveness, and knowledge. Patients are often happy with virtual consultation services when staff members show empathy and clear communication. ^15^ On the other hand, the relatively low satisfaction with the response time and the accuracy of referrals are common challenges in contemporary healthcare call centres. ^14^ Therefore, the skills and responsiveness of employees are integral in the performance of a health call center.

Satisfaction with doctors’ communication during consultations was high, with participants identifying the communication as respectful, transparent, and empathetic. Kruse et al. ^16^ affirm that clear and respectful doctor communication is a major driver of satisfaction in telemedicine contexts. Doctors that engage patients make remote calls effective in healthcare by enhancing clarity of the health issues and care plans they discuss with patients.

The satisfaction ratings with the call center were relatively higher among female respondents. Gendered perceptions of service effectiveness are mostly linked to communication preferences and expectations related to service. ^17^ Hence, customization of the call services to match gendered perceptions is worth exploring to ensure high satisfaction rates across all genders.

The variations of satisfaction levels among participants with various education levels aligns with conclusions by Seguí et al. (2020) that digital literacy levels, awareness, and expectations, which are dependent on education level, determine satisfaction with a healthcare service like teleconsultation. Participants with at least a high school diploma could be having better digital literacy and more knowledge about call centres, translating into better utilization and comprehension of call centres. The low satisfaction ratings among participants with advanced degrees requires further studies.

The gender influence on satisfaction with doctor communication follows a trend of females reporting higher satisfaction rates with verbal empathy and medical explanations that potentially aid in resolving health issues (Downes et al., 2017; Bouman et al., 2017). The low rate of resolving issues through the 151 calls is a limitation of teleconsultation in solving complex cases. Portnoy et al. (2020) noted that telemedicine services are more effective for initial assessments than complete treatment plans, hence a high likelihood of unresolved or stagnated health issues when the call center is tasked with delivering more comprehensive care plans beyond the basic initial assessments.

The Kuwaiti had positive reviews regarding most aspects of the 151 call center. The call center promotes healthcare awareness in the country, saves significant time needed for in-person visits, and reduces unnecessary visits to healthcare facilities (Ohannessian et al., 2020). However, Capozzo et al. ^13^ indicated that many patients prefer in-person visits and do not believe in teleconsultation and telehealth. Additionally, the cross-demographic comparisons in this study revealed gender and education level disparities regarding patient satisfaction with call center services, which were earlier reported by Seguí et al. ^18^ and Tai-Seale et al. ^8^

Participants who had never used the 151-call service in Kuwait cited service unawareness, prior negative call feedback experiences like unanswered calls, and the lack of a perceived need for the service. Inadequate promotion, limited awareness, and unclear messaging impede the usage of telehealth services in many contexts. ^19^ Thus, limited public knowledge is a technology adoption barrier. Despite implementing targeted outreach tools, the limited awareness about Kuwait’s 151 healthcare call center model propagates disengagement, implying that the MOH’s initiatives have been inefficient in disseminating accurate and precise information about the service. This disparity highlights the need for more focused public education initiatives to raise awareness and promote service use.

A key strength of this study is its robust internal consistency, demonstrated by high Cronbach’s alpha values across all the subscales since they exceeded the commonly accepted threshold of 0.70. ^20^ Another strength is the study’s large and diverse sample, which is representative since it included participants from all major governorates in Kuwait. Additionally, the wide range of ages and education levels enhanced the generalizability of the findings in the Kuwaiti context. However, a key limitation is using a non-probabilistic convenience sampling method via social media, which may have introduced selection bias and limited the sample’s representativeness. ^21^ Individuals without internet access or with lower digital literacy may have been excluded, potentially skewing satisfaction ratings.

Continued use of the 151 call center is recommended since users were satisfied with most of its aspects. However, response time and service awareness should be optimized. Future studies should test gender- and education level-customized 151 call center interventions using a pre-test post-test quasi-experimental study design to generate stronger evidence on how the call center services can be improved. A qualitative study is also recommended to investigate reasons for the low satisfaction rates among people with advanced degrees.

## Ethical considerations

The user satisfaction survey conducted in this study anonymously assessed a service rather than humans without collecting any personal identifiers or sensitive data of the respondents, posing minimal risk to the respondents, hence waiver of ethical review was granted by the Health Sciences Center Ethical Committee, Kuwait University, Kuwait. The privacy of the respondents was guaranteed by anonymizing the data collection as defined in the latest issue of the Declaration of Helsinki. All the respondents provided informed consent before answering the survey questions by reading a statement about the purpose of the survey and respondents’ rights including confidentiality of responses and non-obligation to participate on the first page of the survey, then choosing to participate or exit the survey by clicking either of “I agree to participate” or “I choose to exit” buttons.

## Data Availability

The User satisfaction with the 151 call center in Kuwait (XLSX) are available in Mendeley Data: Aldousari E, Kithinji D. User satisfaction with the 151 call center in Kuwait [dataset]. Mendeley Data. 2025; V1. Available from: https://doi.org/10.17632/8854gk66td.1. ^22^ Data are available under the terms of the Creative Commons Attribution 4.0 International license (CC-BY 4.0). The questionnaire that was used for data collection is included in the same repository. ^22^
