# Supplementary material for: Personalizing health call center services: results from an online survey on user experiences in Kuwait
Source: F1000Res. 2026 Mar 5;15:364. [Version 1] doi: 10.12688/f1000research.170997.1 (PMC13550925; doi:10.12688/f1000research.170997.1)
Supplement: Peer review history

# Reviewer report: Reviewer response for version 1

**DOI:** 10.5256/f1000research.188529.r495195

**Published:** 2026-07-08

**Authors:** Nagwa Hamad

**Affiliations:** 1 Nursing, Alexandria University Faculty of Nursing, Alexandria, Alexandria Governorate, Egypt

The manuscript describes recruitment via an online convenience sample (Twitter, WhatsApp, Facebook), which likely under‑represents individuals with lower digital literacy, and those who do not actively use social media. In light of these limitations, I recommend removing the term “nationally representative” from the conclusion.

 Because the study uses a cross‑sectional design, it cannot establish causality. Please revise statements such as “gender and education level influenced satisfaction” to “gender and education level were associated with satisfaction.”

 The eligibility criteria specify inclusion of adults aged 18 years and above; however, Table 1 includes participants younger than 18 years (2.8%). Please remove this category from Table 1 or clarify and correct the eligibility criteria and sample description to ensure consistency.

Is the work clearly and accurately presented and does it cite the current literature?

Yes

If applicable, is the statistical analysis and its interpretation appropriate?

Yes

Are all the source data underlying the results available to ensure full reproducibility?

Yes

Is the study design appropriate and is the work technically sound?

Yes

Are the conclusions drawn adequately supported by the results?

Yes

Are sufficient details of methods and analysis provided to allow replication by others?

Yes

Reviewer Expertise:

Nursing education, research methodology, health services/telehealth quality assurance/pediatric nursing/child psychology/neonatology/critical pediatrics.

I confirm that I have read this submission and believe that I have an appropriate level of expertise to confirm that it is of an acceptable scientific standard.

# Reviewer report: Reviewer response for version 1

**DOI:** 10.5256/f1000research.188529.r468532

**Published:** 2026-04-11

**Authors:** Sally Mohammed Farghaly

**Affiliations:** 1 Alexandria University Faculty of Nursing, Alexandria, Egypt

RequiredMinor Revisions (to be included).

 Methodological clarity:

 Include the questionnaire or main items within the text or an appendix.

 Clarify how statistical assumption testing was carried out for the ANOVA test.

 More details on the missing data section.

 Stronger literature:

 Ensure there are 3-5 years of current references regarding call center satisfaction and telehealth.

 Include more regional studies, if available (Gulf region/Middle East).

 Stronger statistics:

 Include the effect size in reporting alongside the p-value.

 Minor edits to language and format:

 Correct minor grammar errors, typos, or spacing/punctuation issues throughout.

 Consistent decimal use/percentage reporting.

Is the work clearly and accurately presented and does it cite the current literature?

Yes

If applicable, is the statistical analysis and its interpretation appropriate?

Yes

Are all the source data underlying the results available to ensure full reproducibility?

Yes

Is the study design appropriate and is the work technically sound?

Yes

Are the conclusions drawn adequately supported by the results?

Yes

Are sufficient details of methods and analysis provided to allow replication by others?

Partly

Reviewer Expertise:

Health services research, digital health and telemedicine, nursing research and clinical practice, healthcare quality and patient safety, epidemiology and biostatistics, and health systems policy and management.

I confirm that I have read this submission and believe that I have an appropriate level of expertise to confirm that it is of an acceptable scientific standard.
